# Preparation of Temperature Resistant Terpolymer Fracturing Fluid Thickener and Its Working Mechanism Study via Simulation Methods

**DOI:** 10.3390/ma18051171

**Published:** 2025-03-06

**Authors:** Bo Zhang, Bumin Guo, Guang’ai Wu, Shuan Li, Jinwei Shen, Susu Xing, Yujie Ying, Xiaoling Yang, Xinyang Zhang, Miaomiao Hu, Jintang Guo

**Affiliations:** 1 China Oilfield Services Limited, Tianjin 300450, China; zhangbo21@cnooc.com.cn (B.Z.); guobm@cnooc.com.cn (B.G.); lishuan@cnooc.com.cn (S.L.); shenjw5@cnooc.com.cn (J.S.); 2 CNOOC Beijing Research Center, Beijing 100027, China; wuga@cnooc.com.cn; 3 School of Chemical Engineering and Technology, Tianjin University, Tianjin 300350, Chinammhu1990@tju.edu.cn (M.H.); 4 Zhejiang Institute of Tianjin University, Shaoxing 312300, China; 5 Haihe Laboratory of Sustainable Chemical Transformations, Tianjin 300192, China

**Keywords:** hydrophobic association, molecular simulation, thickener, thermostable performance

## Abstract

To enhance oil and gas recovery, a novel hydrophobic terpolymer was synthesized via free radical polymerization. The terpolymer consists of acrylamide, acrylic acid, and hydrophobic monomers, and is used as a hydraulic fracturing fluid thickener for freshwater environments. Hydrophobic groups were introduced into terpolymer to improve its tackiness and temperature resistance. The conformation and key parameters of hydrophobic monomers at different temperatures were investigated through a combination of experiments and molecular dynamics simulations. These methods were employed to elucidate the mechanism behind its high-temperature resistance. The experiment results show that, at concentrations between 0.2% and 0.4%, significant intermolecular aggregation occurs, leading to a substantial increase in solution viscosity. Configuring the base fluid of synthetic polymer fracturing fluid with 1% doping, the apparent viscosities of the base fluid were 129.23 mPa·s and 133.11 mPa·s, respectively. The viscosity increase rate was 97%. The base fluid was crosslinked with 1.5% organozirconium crosslinker to form a gel. The controlled loss coefficient and loss velocity of the filter cake were C_3_ = 0.84 × 10^−3^ m/min^1/2^ and v_c_ = 1.40 × 10^−4^ m/min at 90 °C, meeting the technical requirements for water-based fracturing fluid. Molecular dynamics simulations revealed that the radius of gyration of the hydrophobically linked polymer chain segments decreases as the temperature increases. This is due to the increased thermal motion of the polymer chain segments, resulting in less stretching and intertwining of the chains. As a result, the polymer chains move more freely, which decreases the viscosity of the solution. In conclusion, the proposed fracturing fluid thickener system demonstrates excellent overall performance and shows significant potential for application in oil and gas recovery.

## 1. Introduction

Hydraulic fracturing technology, initially developed in the mid-20th century to alleviate construction-related blockages, has evolved into a primary method for enhancing oil and gas production. This technology involves the creation of fractures in reservoir rocks to facilitate the flow of hydrocarbons. As exploration ventures into deeper and more extreme conditions, the development of fracturing fluids capable of withstanding high temperatures and salinities has become increasingly critical [1].

Among them, thickener is one of the key materials for fracturing fluid [2]. Currently, there are three types of water-based fracturing fluids used at home and abroad: natural plant gums, synthetic polymers [3], and viscoelastic surfactants [4]. Taking guanidine gum [5] as an example, it has high viscosity, good thickening ability, and strong shear resistance. However, it is difficult to break through the ultra-high temperature limit, and this cannot completely break the glue. Throughout this process, the residual content is high and the formation is seriously damaged. Although clean fracturing fluids are easy to break the rubber, the residue content after breaking the rubber is small, and the damage to the formation is small among other advantages. There are also problems including high cost, large usage, poor temperature resistance, and filter loss reduction. Cellulose fracturing fluids [6,7,8] have a large amount of residue, poor salt resistance, and water solubility, and it is difficult to recycle the return fluid. The above fracturing fluids’ high cost, poor stability, high molecular resistance and energy consumption of the mixed solution, poor adaptability to the downhole temperature and environment, low return rate, high residue, and serious secondary damage to the reservoir, have hindered their wide application. Compared with the above fracturing fluids thickener, polymer-based fracturing fluids thickeners have a controllable structure and are more widely used due to their enhanced temperature and salt resistance [9,10,11,12,13,14]. Cao et al. [10] investigated the synthetic polymer, poly-(acrylamide-co-acrylic acid-co-2-acrylamido-2-methyl-1-propanesulfonic acid) (P3A), as a rheology modifier for water-based fracturing fluids under high-temperature and high-salt conditions, and compared it with the guar gum system. The results showed that the thickening ability of P3A was significantly better than that of guar gum. Wu et al. [11]. prepared hydrolyzed polyacrylamide (HPAM)-based and GG-based fracturing fluids using guar gum (GG) and polyacrylamide (HPAM) as tackifiers, respectively, and evaluated their damage to the core. It was found that the molecular size of the residual polymer in the HPAM-based system was smaller than that in the GG-based system, for the same viscosity as that of the gel fracturing fluid.

The most widely used synthetic polymer thickener is HPAM, which has excellent water solubility [15]. However, the viscosity of HPAM solutions decreases with increasing mineralization and temperature, which limits the use of HPAM in high temperature and high mineralization reservoirs [16,17]. To improve the temperature resistance of HPAM solutions, functional groups can be introduced through molecular design to improve the water solubility, temperature resistance, and shear resistance of polymers [14].

The temperature resistance of polymer thickeners can be improved by introducing hydrophobic monomers into the polymer backbone [18,19,20]. The aggregation of the hydrophobic groups of the polymer occurs as a result of hydrophobic interaction, resulting in intramolecular and intermolecular linkages in the macromolecular chain [21]. When the polymer concentration is higher than a certain critical concentration (CAC), the macromolecular chains are hydrophobically aggregated to form a supramolecular structural dynamic physical crosslinking network dominated by intermolecular aggregation. As the hydrodynamic volume increases, the viscosity of the solution increases sharply. In addition, the polymer introduces both cations and anions, forming an electrostatic bridge in water and improving the shear resistance of the system. However, the viscosity of the aqueous polymer solution decreases with increasing temperature. This can be attributed to the change in the chain conformation of the polymer as the temperature increases. For polymer solutions, the chain conformation is the result of the interaction between the monomer and solvent molecules [22]. Therefore, understanding the conformational changes in hydrophobically linked polymers alongside their temperature is beneficial to reveal the mechanism of their temperature resistance.

In conclusion, a ternary amphiphilic statistical copolymer thickener for the high temperature fracturing system was prepared by using acrylamide and acrylic acid as the main chains, and by introducing cetyl methyl diallyl ammonium chloride (C_16_DMAAC). Using a combination of experiments and molecular dynamics simulations, the conformations and key parameters of the hydrophobic monomers at different temperatures were discussed in depth, and the mechanism of their high-temperature resistance was elaborated, which provides a theoretical basis for the subsequent polymerization of this type of monomer.

## 2. Materials and Methods

### 2.1. Materials

Acrylic acid (AA) and sodium hydroxide (NaOH) were supplied from Tianjin JiangTian Chemical Industry Co., Ltd. (Tianjin, China). Acrylamide (AM) was procured from Shandong Baomo Biochemical Co., Ltd. (Dongying, China). Ammonium persulfate (APS) was bought from Dibo Biotechnology Co., Ltd. (Shanghai, China). Sodium bisulfite was provided by Tianjin Damao Chemical Reagent Co., Ltd. (Tianjin, China). Lastly, Cetyl methyl diallyl ammonium chloride (C_16_DMAAC) was purchased from Hubei Shiteng Chemical Technology Co., Ltd. (Jingmen, China). Organozirconium crosslinker was prepared by China Oilfield Services Limited, (Tianjin, China).

### 2.2. Synthesis of Terpolymer Thickener

**Figure 1:**
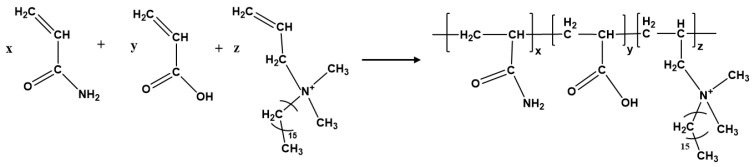
The synthesis procedure for the MAC terpolymer.

Utilizing an analytical balance, measure specific ratios of AM, AA, and C_16_DMAAC into a 500 mL beaker, and then introduce a predetermined volume of distilled water to prepare a homogeneous mixture. Among them, the mass ratio of AM: AA: C_16_DMAAC is 5:1:0.05. Thoroughly mix and agitate the solution before placing it in a temperature-controlled water bath. Introduce an inert gas into the system for 30 min to expel dissolved oxygen from the solution. Once the solution reaches the initiation temperature for polymerization, add a predetermined quantity of a redox initiator to commence the polymerization reaction. Continue to purge the solution with inert gas until it becomes viscous, and then seal the beaker with plastic film. Transfer the sealed beaker back into the temperature-controlled water bath and allow the reaction to proceed for 2 h until the polymerization is complete, resulting in a gel-like substance. Remove the gel block from the beaker, immerse it in anhydrous ethanol for several hours to remove any unreacted monomers, and then cut the gel into smaller pieces for granulation to obtain the desired polymeric fracturing fluid thickening agent (AM-AA-C_16_DMAAC terpolymer) and name it MAC. The schematics of the preparation process of the MAC terpolymer are shown in Figure 1.

### 2.3. Characterization of Terpolymer Thickener

The chemical structure and elemental analysis of the purified MAC terpolymer were analyzed using Fourier-transform infrared spectroscopy (FTIR, iS50, Thermo, Waltham, MA, USA, 500 cm^−1^ to 4000 cm^−1^) and ^1^H Nuclear Magnetic Resonance (NMR, AVANCE III, Bruker, Fällanden, Switzerland). The micromorphology of these samples was measured by scanning electron microscopy (SEM, Regulus 8100, Hitachi, Tokyo, Japan) and an Atomic Force Microscope (AFM, Dimension icon, Bruker, Billerica, MA, USA). The sample preparation method is as follows: Firstly, the polymer of different qualities was dissolved in the aqueous solution, and the fracturing fluid base fluid was obtained after standing for 4 h. Then, the base solution was freeze-dried so that the polymer retained its structural state in an aqueous solution. Finally, a small amount of dried sample was placed on the conductive adhesive for SEM testing. The thermal stability of the terpolymer was assessed using a Thermogravimetric analysis (TGA, TG 209F3, Netzsch, Selb, Germany) under a nitrogen atmosphere, with a temperature ramp of 10 °C/min from 35 °C to 600 °C. Dynamic light scattering methods were used to assess the size of polymer solution micelle particle size using a zeta potential and intensity analyzer (DLS, Zetasizer Nano ZS, Malvern, UK).

Fracturing fluid performance testing was conducted under the People’s Republic of China natural gas industry standard SY/T 7627-2021 [23]: Technical requirements for water-based fracturing fluids. The apparent viscosity of the polymer base fluid was evaluated by using a HTD-12DST rotational viscometer (Hangzhou, China). The methodology is as follows: A synthetic polymer fracturing fluid base fluid was prepared with a blend of 1%. The prepared fracturing fluid base fluid sample was transferred to the measuring cup of a rotational viscometer. Then, the apparent viscosity μ was measured by the rotational viscometer at a rotational speed of 100 r/min (shear rate of 170 s^−1^), which was read after the needle of the viscometer stabilized and was recorded as α. The measurements were repeated three times according to the above operation, and the average value was taken. Apparent viscosity is calculated according to the following Formula (1):

(1) $$\mu =\frac{5.077\alpha }{1.704}\;.$$

In the formula:

μ—Apparent viscosity of the sample, (mPa·s);
α—100 r/min-Viscometer pointer reading at RPM;
5.077—Value of shear stress when α is 1, (10^−1^ Pa);
1.704—Value of shear rate for a viscometer rotation of 1 r/min, (s^−1^).

The rate of viscosity increase is calculated by the following Equation (2):

(2) $$\gamma =\frac{\eta_{1}}{\eta_{2}}\times 100\%.\;$$

In the formula:

γ—Viscosity increase rate, expressed as a percentage;
η_1_—Viscosity value at the end of specimen preparation, (mPa·s);
η_2_—Viscosity value of the specimen when resting for 4 h, (mPa·s).

Take 100 mL of the base liquid sample, stir well with a glass rod, add 1.5% crosslinking agent, stir rapidly with a glass rod and time, and record the crosslinking time needed to form a pick hanging gel.

The static filtration loss performance of the fracturing fluid was tested by a high temperature and high-pressure filtration loss meter at a temperature of 90 °C, an initial pressure of 0.69 MPa, and a differential pressure of 3.50 MPa. The cumulative filtration loss of the filtrate was recorded for 1 min, 2 min, 4 min, 9 min, 16 min, 25 min, and 36 min, respectively [24]. Plot the cumulative filtration loss on the *y*-axis and the square root of time on the *x*-axis. The cumulative filtration loss is linearly related to the square root of time.

The loss coefficient C_3_, which is controlled by the filter cake, and the loss velocity v_c,_ are calculated by the following Equations (3) and (4):

(3) $$C_{3}=0.05\times \frac{m}{A}\;,$$

(4) $$v_{c}=\frac{C_{3}}{\sqrt{t}}\;.\;$$

In the formulas:

C_3_—Cake control loss coefficient, (m/min^1/2^);
m—Slope of the filtration loss curve, (mL/min^1/2^);
A—Filter loss area, (cm^2^);
v_c_—Filtration rate, (m/min);
t—Filtration time, (min).

## 3. Results and Discussion

### 3.1. Characterization of Terpolymer

#### 3.1.1. FTIR Analysis of MAC

**Figure 2:**
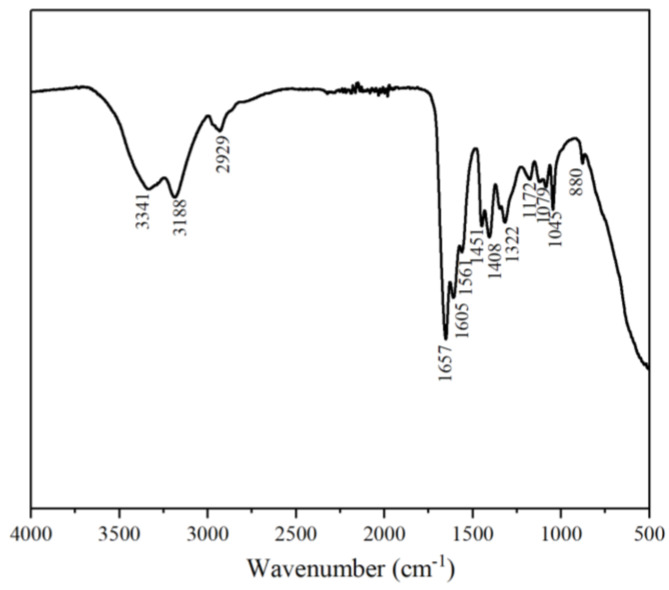
FTIR spectrum of the hydrophobic associative polymer MAC.

The FTIR spectrum of the hydrophobically bound polymer MAC is shown in Figure 2. The stretching vibrational absorption peak of the N-H of the amide group is at 3341 cm^−1^, and the stretching vibrational absorption peak of O-H is located at 3188 cm^−1^. The stretching vibrational absorption peaks of the methyl and methylene groups are near 2929 cm^−1^. The position of the stretching vibrational absorption peak of C=O is at 1657 cm^−1^, and 1605 cm^−1^ and 1561 cm^−1^ are the bending vibrational absorption peaks. The characteristic absorption peak for methylene is at 1451 cm^−1^. 1322 cm^−1^ is the asymmetric stretching vibrational absorption peak of C-H when -CH_3_ is coupled to a heteroatom. 1172 cm^−1^ is the C-O stretching coupled O-H in-plane bending absorption peak. The C-CH_2_ stretching vibrational absorption peak is at 1079 cm^−1^ for long chain alkyl groups. The C=O bending vibrational absorption peak of the carboxyl group is at 880 cm^−1^. The presence of amide, carboxyl, and alkyl groups in the molecular chain can be seen from the plots, indicating that hydrophobic groups were introduced into the molecular chain. By analyzing the infrared spectrogram of the hydrophobically modified terpolymer, it was determined that it conformed to the theoretical structure and was the target polymer [25,26,27].

#### 3.1.2. ^1^H NMR Analysis

**Figure 3:**
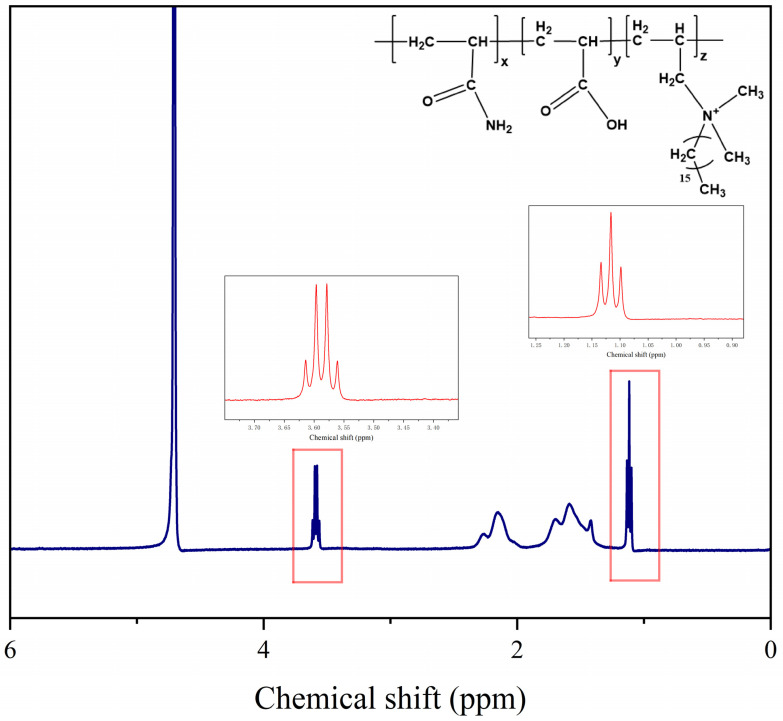
^1^H NMR spectra of polymer MAC.

The ^1^H NMR spectrum (400 MHz, D_2_O) of polymer MAC is shown in Figure 3. It can be seen from the figure that the peaks between 1.11 ppm belong to the proton peaks of -CH_3_ on the long chain alkyl group of C_16_DMAAC. The proton peak of the methylene group on the main chain and the long-chain alkyl of the copolymer C_16_DMAAC is at 1.67 ppm. The peaks between 2.0 and 2.5 ppm belong to the -CH protons. The peak at 3.57 ppm is assigned to the proton peak of the methylene group linked to the nitrogen atom on the C_16_DMAAC. The peak at 4.7 ppm is attributed to the introduction of solvent deuterated water molecules. The successful participation of C_16_DMAAC in copolymerization is evident from the NMR hydrogen spectroscopy results [28,29].

#### 3.1.3. GPC Analysis

The molecular properties of the synthesized MAC polymers were evaluated by the GPC test. The molecular parameters are given in Table 1. In Table 1, the molecular weight (Mw) of the synthesized polymers is 1,190,046 g/mol.

### 3.2. Investigation into the Mechanism of Hydrophobic Association of Polymer MAC in Aqueous Solutions

#### 3.2.1. SEM Analysis

**Figure 4:**
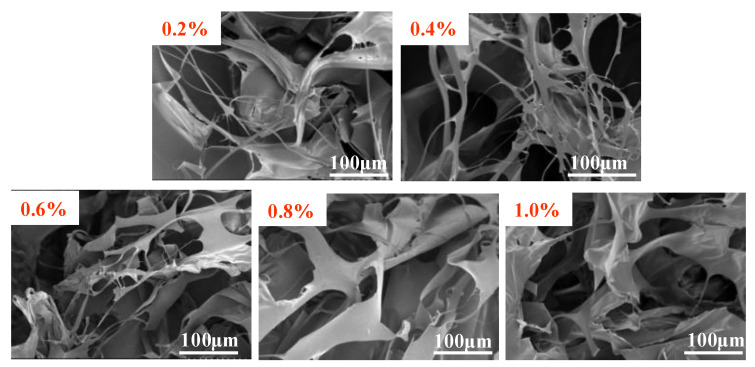
SEM images of ternary amphiphilic statistical copolymer MAC in distilled water at different mass concentrations.

The microscopic morphology of hydrophobically conjugated polymer MAC in freshwater at different mass concentrations was observed with the aid of field emission electron microscopy, as shown in Figure 4, which preliminarily revealed the change in the microstructure of the polymer from a low concentration to a higher concentration. Due to the existence of intramolecular and intermolecular associations in the hydrophobically bound polymer solution, the hydrophobically bound polymer exists in the solution in the form of aggregates and is not dispersed in the solution in a single molecular state. After the polymer solid particles are dissolved in water, they dissolve into loose network structures, which are mainly centered on larger aggregates. At a mass concentration of 0.2%, the polymers mostly appear in a nematic state, where it is tentatively inferred that chain entanglement occurs. At a mass concentration of 0.4%, the polymer wire bundles are interconnected to form a network structure. As the concentration increases, the number of polymer aggregates per unit area increases, and a chain entanglement between the polymers exists. The individual dispersed structures form a stronger network structure through hydrophobic linkage. However, at too high a concentration, extensive gelation occurs, and the polymer can form a gel-like structure.

#### 3.2.2. AFM Analysis

**Figure 5:**
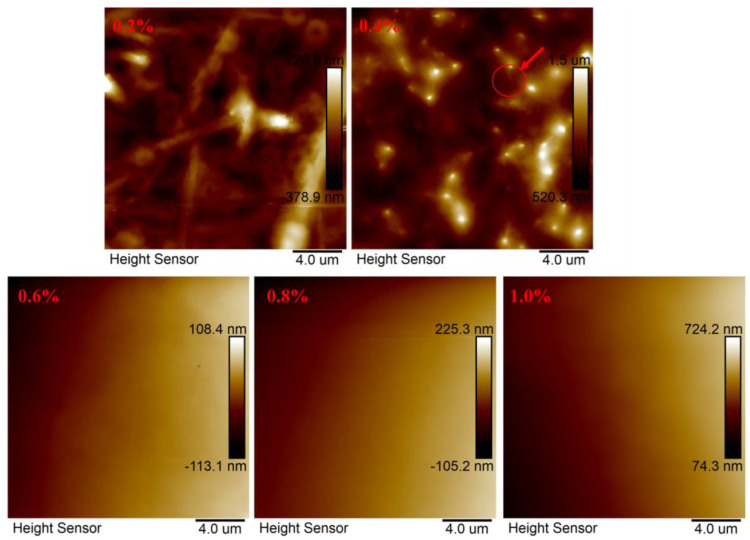
AFM plots of hydrophobically bound polymer MAC in distilled water at different mass concentrations.

Through observing the microscopic morphology of ternary amphiphilic statistical copolymer MAC in distilled water at different mass concentrations, as shown in Figure 5, the change in the microstructure of the polymer, from a low concentration to a higher concentration, was initially revealed. At a mass concentration of 0.2%, the polymer exhibits a nematic state and a cyclic structure. This is due to the in situ formation of loose network structures when the polymer solid particles are dissolved in water, and these structures are mainly centered on larger aggregates. At a mass concentration of 0.4%, the number of polymer aggregates per unit area increases. The polymers are interconnected in a “dendritic” fashion, centered on larger aggregates. The dispersed structures form a network structure through hydrophobic interactions. The surface of the polymers at concentrations of 0.6%, 0.8%, and 1% by mass is flat and unchanged. This is due to the high concentration, wherein a large-area gelation phenomenon occurs, and the polymer forms a gel-like structure, which is consistent with the SEM results [30].

#### 3.2.3. TGA Analysis of MAC

**Figure 6:**
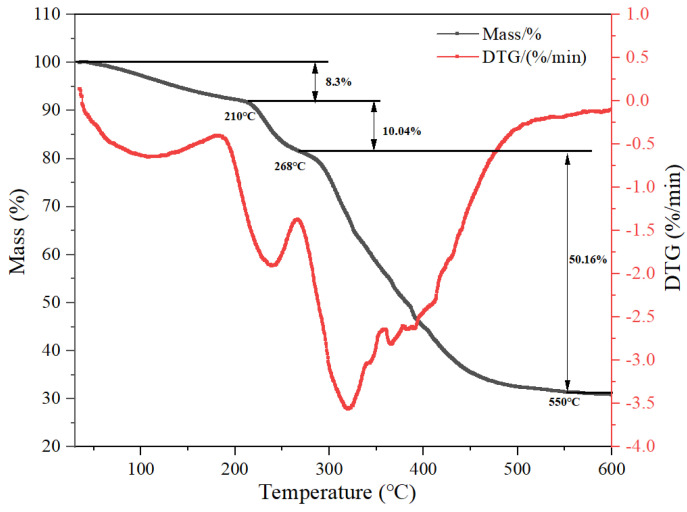
TGA curves of the hydrophobic associative polymer MAC.

It was found through thermogravimetric analysis, as shown in Figure 6, that the decomposition temperature of the polymer reflects the thermal stability of its macromolecular chain groups. The TGA curves of the polymers showed the following phases of weight loss. The initial phase occurs between 30 to 210 °C and is mainly attributed to weight loss due to evaporation of intermolecular water. The weight loss at this stage is the result of two factors, namely the evaporation of a large amount of free water between 30 and 120 °C, and the evaporation of bound water associated with amide and methylene groups in the temperature range of 120 to 210 °C. The second stage of decomposition occurs in the temperature range of 210–268 °C and is mainly attributed to the decomposition of the amide groups within the polymer. The cause of weight loss between 268 °C and 550 °C is mainly due to the thermal decomposition of the polymer chain segments. This indicates that the polymer is stable at around 200 °C.

#### 3.2.4. Dynamic Light Scattering (DLS)

The relationship between different concentrations and micelle particle sizes was studied using DLS. The results are shown in Table 2.

According to Table 2, the particle size of the polymer at 0.2% was 2871 nm. Due to the greater dispersion of the polymer in the low concentration solution, the hydrophobic chains of the polymer are mainly intramolecularly bound, and the polymer particle size is smaller. The polymer particle size at 0.4% is 5811 nm, which is significantly higher than that at 0.2%. This is mainly due to intermolecular polymerization and chain entanglement. The particle size of the polymer at 0.6% was 7475 nm. The increasing polymer concentration leads to a decrease in the distance between polymer molecules. Intermolecular linkages between polymer chains occur and form dynamic physical crosslinked networks, which lead to a dramatic increase in the mechanical volume and viscosity of the fluid. This is consistent with the viscosity–concentration curve results.

### 3.3. Properties of MAC Aqueous Solution

#### 3.3.1. Basic Properties

The basic performance parameters of the ternary amphiphilic statistical copolymer thickener MAC are shown in Table 3.

#### 3.3.2. Apparent Viscosity and Viscosity Increase Rate

A synthetic polymer fracturing fluid base fluid was prepared with a blend of 1%. The test results of the apparent viscosity of the base fluid are shown in Table 4.

The analysis of the results yields that the viscosity increase rate is 97%, which is greater than 80% and meets the technical requirements of the project.

#### 3.3.3. Effect of Concentration on the Apparent Viscosity of Polymers

**Figure 7:**
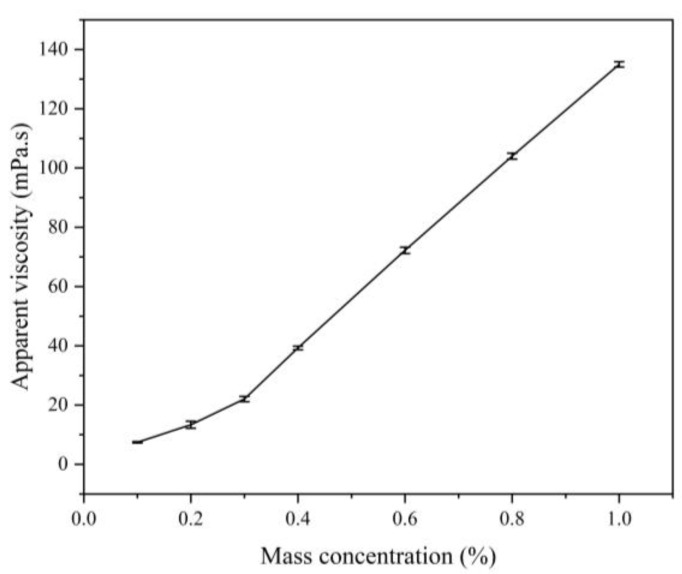
Effect of different mass concentrations on the apparent viscosity of the polymer MAC solutions.

The aggregation of hydrophobic groups of polymers occurs due to hydrophobic interactions, resulting in intramolecular and intermolecular associations of macromolecular chains. When the polymer concentration is higher than a certain critical concentration (CAC), the macromolecular chains are aggregated through hydrophobic aggregation, forming a supramolecular structure dominated by an intermolecular aggregation dynamic physical crosslinking network, in which the hydrodynamic volume increases and the viscosity of the solution rises dramatically. Figure 7 shows the viscosity–concentration relationship curve of the associative polymer MAC in fresh water. From the figure, it can be seen that when the mass concentration of polymer MAC is lower than 0.3%, the apparent viscosity of the solution of the associative polymer MAC increases linearly with the increase in the polymer concentration. Also, the increase in the apparent viscosity of the solution becomes larger after it is higher than the polymer concentration. This indicates that the critical binding solubility of hydrophobic binding polymer MAC in freshwater is near 0.3%.

#### 3.3.4. The Relationship Between Temperature and Apparent Viscosity

**Figure 8:**
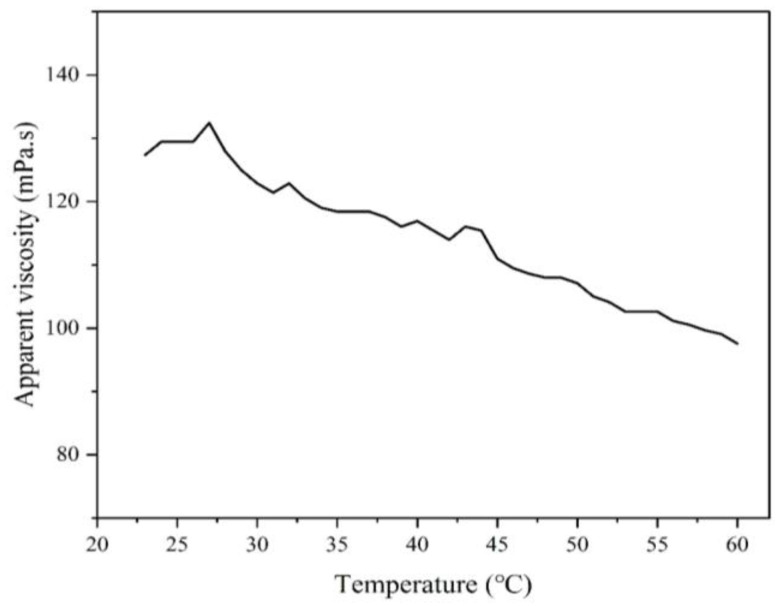
The influence of temperature on the apparent viscosity of polymer solutions.

To investigate the temperature-dependent rheological properties of the polymer solution, a 1 wt% MAC solution was prepared, and its apparent viscosity was measured at a shear rate of 170 s^−1^ over a temperature range from 20 °C to 60 °C. Figure 8 illustrates the observed changes in viscosity with the increase in temperature.

Initially, the apparent viscosity showed a slight increase with temperature. This behavior can be attributed to the rise in system entropy, which enhances intermolecular associations. Additionally, the temperature-induced extension of molecular chains promotes further intermolecular interactions. However, as the temperature continued to rise, the apparent viscosity exhibited a noticeable decrease. This reduction is likely caused by intensified thermal motion, which disrupts the hydrophobic interactions between polymer groups and water molecules. Consequently, the hydrophobic association weakens, leading to a reduced fluid mechanics volume and a decline in viscosity.

These findings provide insight into the interplay between molecular interactions and thermal effects, offering valuable data for applications requiring the precise control of polymer solution rheology under varying thermal conditions.

#### 3.3.5. Temperature and Shear Resistance Performance

**Figure 9:**
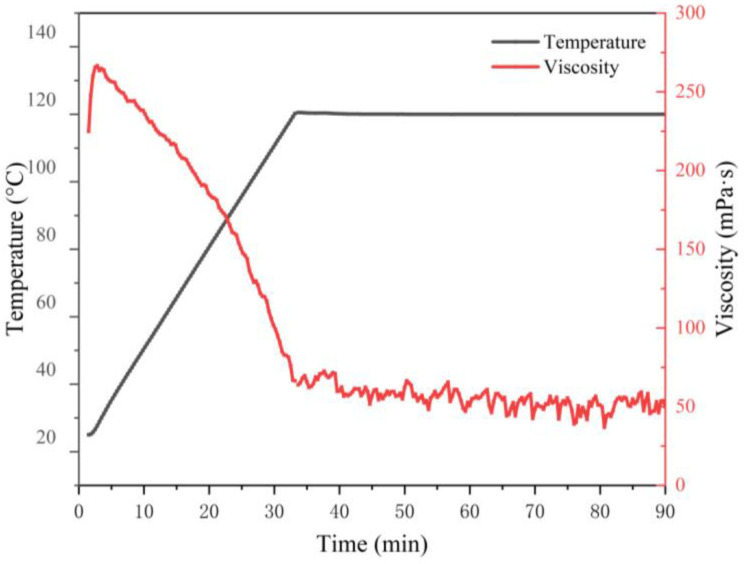
Shear resistance of 1% polymer MAC solutions at 120 °C.

According to the People’s Republic of China natural gas industry standard SY/T 7627-2021: Technical requirements for water-based fracturing fluids, the fracturing fluid with higher viscosity and no cross-linking formed by polymer as thickener, water as solvent, and the cross-linking agent, is called linear gel fracturing fluid. Its temperature and shear resistance are required to be ≥20 mPa·s. Testing the temperature resistance and the shear resistance of polymer solutions is necessary to initially assess their compliance with the requirements of linear gel fracturing fluids. We tested the temperature and shear resistance of a polymer solution with a dosage of 1% at 120 °C using a high-temperature and high-pressure rheometer. As shown in Figure 9, the viscosity of the terpolymer first increases slightly and then decreases with the increase in shear time. In the early stage of shearing, the macromolecular chains are stretched under the action of shearing, thereby increasing intermolecular binding. As the shearing time progresses, some molecular chains break, the physical crosslinked network structure is disrupted, and the viscosity decreases [31]. The rate of decline is most pronounced in the initial stage and then becomes milder. After 90 min, the viscosity of the polymer solution remained at around 50 mPa·s. This indicates that the hydrophobic polymer MAC solution has good temperature and shear resistance.

### 3.4. Properties of MAC Fracturing Fluid System

#### 3.4.1. Temperature and Shear Resistance Performance

**Figure 10:**
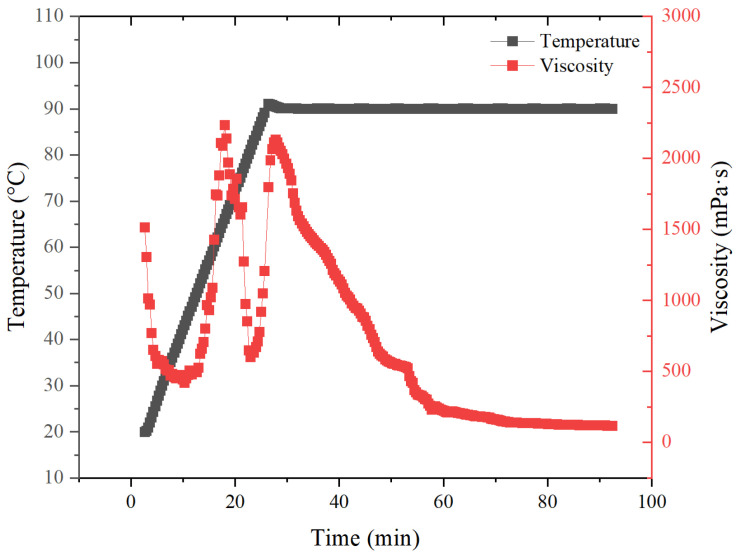
Shear resistance of 1% polymer MAC crosslinked lyophilized gel system at 90 °C.

The temperature and shear resistance of polymer crosslinked jellies doped with 1% were tested at 90 °C using a high temperature and high pressure rheometer. As shown in Figure 10, the multipoint value taking method was used to determine the temperature and shear resistance of the fracturing fluid. The results show that at 90 °C, the viscosity of the polymer solution remains around 118 mPa·s. Compared with the performance of conventional guanidine gel fracturing fluid [32], its apparent viscosity is significantly higher than that of the guanidine gel fracturing fluid, so the temperature resistance of MAC fracturing fluid is better than that of GG fracturing fluid.

#### 3.4.2. Crosslinking Time

The polymer MAC base solution is prepared according to the People’s Republic of China natural gas industry standard SY/T 7627-2021: Technical requirements for water-based fracturing fluids. Firstly, take 100 mL of base solution, add 1.5% organozirconium crosslinking agent, and stir rapidly with a glass rod and time. The time needed to form a pick hanging gel was recorded as the crosslinking time.

At room temperature, a short period cannot form a particularly good pick hanging gel. At 90 °C, the crosslinking time of the freshwater prepared base fluid sample was 40~160 s. According to the Petroleum and Natural Gas Industry Standard of the People’s Republic of China (SY/T 7627-2021), the crosslinking time of the prepared base fluid meets the technical requirements of water-based fracturing fluids, and the crosslinking time is 30~180 s at 60 °C ≤ T < 120 °C.

#### 3.4.3. Viscoelasticity

**Figure 11:**
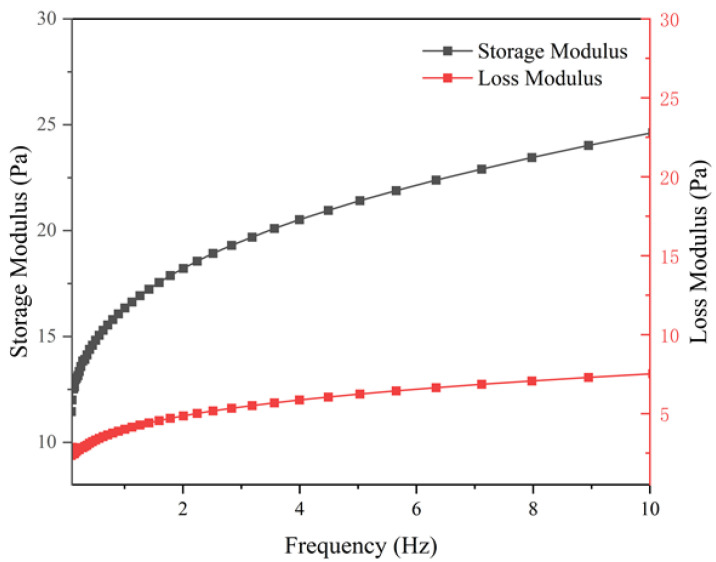
Relationship between modulus and frequency of fracturing fluids doped with 1% hydrophobically bound polymer MAC.

The viscoelasticity of the fracturing fluid doped with 1% hydrophobically conjugated polymer MAC was tested under room temperature conditions. The relationship between its energy storage modulus, loss modulus, and frequency is shown in Figure 11. The analysis of the results reveals that both the energy storage modulus and loss modulus of the MAC fracturing fluid increase with frequency, and the energy storage modulus is always greater than the loss modulus. Compared with the results in the literature, it is found that the energy storage modulus and loss modulus of the MAC fracturing fluid are larger than the values of the guanidine gel fracturing system [33]. It indicates that the MAC fracturing fluid has better viscoelasticity and can meet the sand-carrying capacity of fracturing fluid. Compared with guar gum [33], the MAC crosslinking system had better viscoelastic properties, which could meet the sand carrying capacity of fracturing fluid.

#### 3.4.4. Static Loss Performance

The static filtration loss performance of fracturing fluid was tested by a high temperature and high-pressure filtration loss meter at a temperature of 90 °C, an initial pressure of 0.69 MPa, and a differential pressure of 3.50 MPa. The cumulative filtration loss of the filtrate was recorded for 1 min, 2 min, 4 min, 9 min, 16 min, 25 min, and 36 min, respectively [24]. The data were as follows (Table 5):

By calculation, C_3_ = 0.84 × 10^−3^ m/min^1/2^; v_c_ = 1.4 × 10^−4^ m/min.

The static loss of filtration performance of the prepared base fluid meets the technical requirements of water-based fracturing fluids.

#### 3.4.5. Residue Content

Ammonium persulfate was used as a gel breaker at 90 °C. According to the People’s Republic of China natural gas industry standard SY/T 7627-2021: Technical requirements for water-based fracturing fluids, the residual content of the fracturing fluid system was tested. The results are listed in Table 6.

It can be seen from Table 4 that the residual content of the crosslinked MAC system after gel breaking was small, which indicates that the system used as fracturing fluid system was a low damage fracturing fluid.

#### 3.4.6. Compatibility

**Figure 12:**
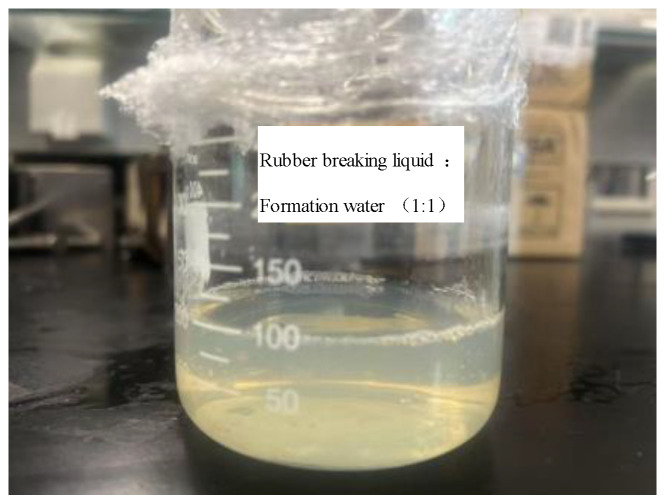
Compatibility of fracturing fluid system with formation water.

The rubber breaking liquid and formation water (4000 mg/L) were taken in a beaker at the volume ratio of 1:1 to form 100 mL mixed liquid. The test temperature was 95 °C, and the results are shown in Figure 12. There was no precipitation or flocculation in the mixture. It shows that the fracturing fluid system has good compatibility with the formation water.

### 3.5. Effect of the Introduction of Hydrophobic Monomers on the Conformation of Polymer Chains

The viscosity of an aqueous solution of a polymer decreases as the temperature increases. This can be attributed to the change in chain conformation of the polymer as the temperature increases. For polymer solutions, the chain conformation is the result of the interaction between the monomer and solvent molecules. Therefore, investigating the effect of hydrophobic monomers on the changes in conformation and key parameters of hydrophobically linked polymers at different temperatures helps reveal the mechanism of their temperature resistance.

**Figure 13:**
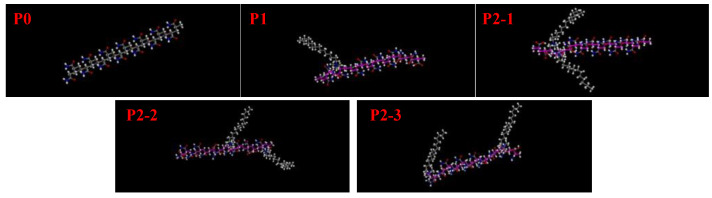
Polymer chain segments contain long chains of different hydrophobic monomers.

The systems were constructed and calculated by molecular dynamics methods using Materials Studio 2017 software [34]. The mechanisms of the introduction of hydrophobic monomers on the temperature resistance of polymers in freshwater and saltwater environments were simulated at 298 K and 433 K, respectively. Five polymer chain segments were constructed by controlling the number and relative positions of hydrophobic monomers, and simulations were performed to investigate the effects of the number (concentration) and positions of hydrophobic groups on the polymer chain segments’ configurations and molecular associations. The five polymer chain segments constructed are shown in Figure 13 as P0, P1, P2-1, P2-2, and P2-3. P0 has no hydrophobic monomer long chain, P1 has one hydrophobic monomer long chain, P2-1 has two hydrophobic monomer long chains, and two chain segments are close to each other. P2-2 has two hydrophobic monomer long chains, and two chain segments are further apart. P2-3 has two hydrophobic monomer long chains, and two chain segments are even farther away from each other.

The model was constructed as follows: AM, AA, and hydrophobic monomer (C_16_DMAAC) were, respectively, modeled to form polymerization units. Polymerization unit 20 was set up and random copolymerization was carried out in a certain proportion to construct polymer chain segments. Geometry optimization was carried out under the COMPASS force field. Then, the optimized polymer and water molecules were filled in the Amorphous Cell Constituent molecules. The periodic box system was constructed, and the polymer water box was annealed five times at 300 K. Finally, the polymer solution model was obtained. Dynamics was selected in the Forcite module for simulation. The force field parameters were the same as before, and the precision was fine. The NVT ensemble was adopted, and the simulation time was 2000 ps.

#### 3.5.1. Effect of Hydrophobic Monomers on the Molecular Conformation of Ternary Polymers at Different Temperatures

**Figure 14:**
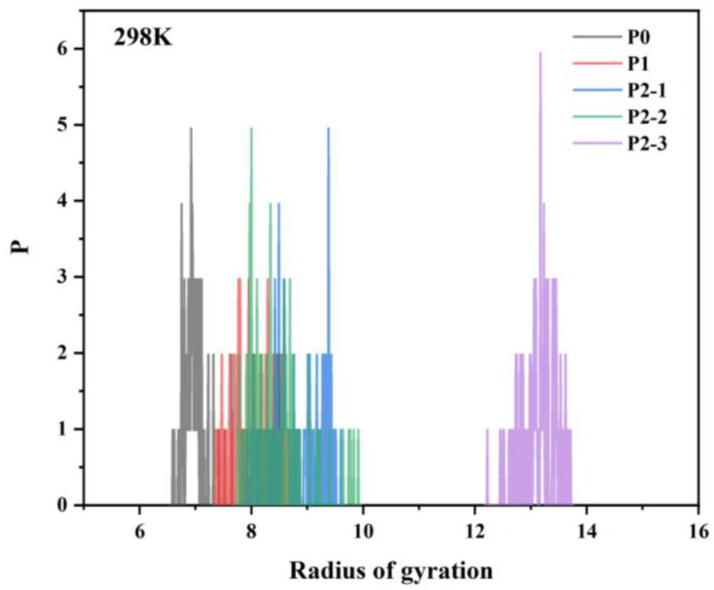
Plot of radius of gyration at 298 K for polymer chain segments containing different hydrophobic monomers.

**Figure 15:**
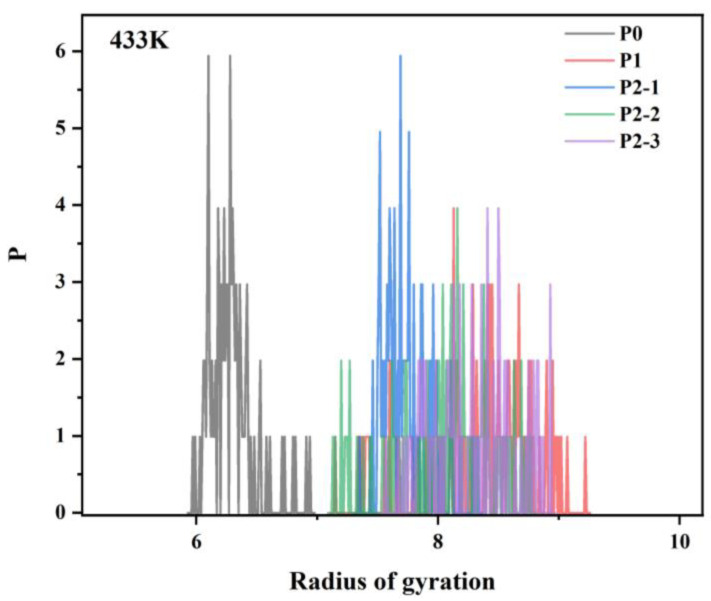
The radius of gyration plots at 433 K for polymer chain segments containing different hydrophobic monomers.

The simulation results are shown in Figure 14 and Figure 15, where the radius of gyration gradually increases with the addition of hydrophobic monomers. It shows that there is an intermolecular association between chain segments with multiple hydrophobic groups, and the intermolecular association is different depending on the relative positions of the polymer chain segments. In the chain segments with multiple hydrophobic groups, there are enough relative positions between the hydrophobic groups to make the intermolecular association stronger, which is conducive to the increase in system viscosity. Simulations revealed that the radius of gyration of the hydrophobically linked polymer chain segments decreases with increasing temperature. This can be attributed to the increased thermal movement of the hydrophobic polymer chain segments at higher temperatures, resulting in less stretching and intertwining of the molecular chains. This results in freer movement of the polymer chains, which reduces the viscosity of the solution. As a result, the viscosity of hydrophobically bound polymer solutions typically decreases at elevated temperatures.

#### 3.5.2. Effect of AA Monomer on the Molecular Conformation of Ternary Polymer at Different Temperatures

The mechanisms of the effect of introducing the AA monomer on the temperature resistance of polymers were simulated in freshwater environments at temperatures of 298 K and 473 K, respectively. Four polymer chain segments were constructed by controlling the number of AA monomers, and their simulations were calculated and analyzed to investigate the effect of the number (concentration) of AA groups on the polymer chain segment conformation and molecular aggregation. The four polymer chain segments without functional monomers were constructed as aa1/aa2/aa3 and aa4 with AA monomer molar ratios of 15%/30%/45% and 60%, respectively.

**Figure 16:**
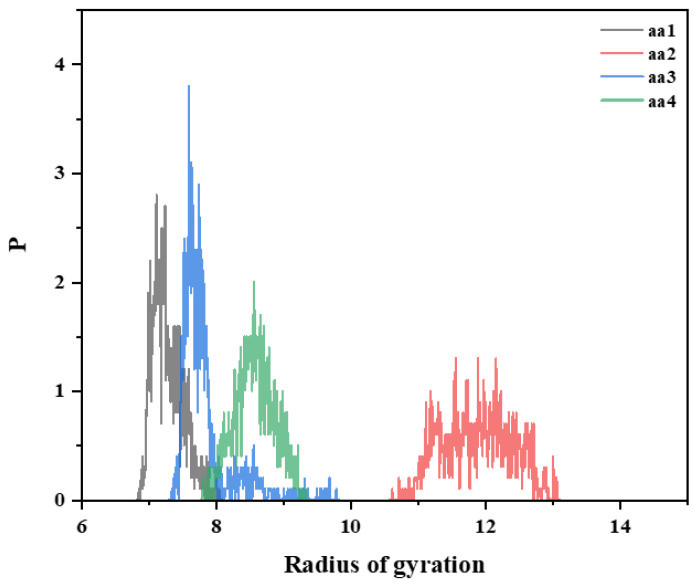
Radius of gyration R_g_ plots of polymer chain segments containing different AA monomers at 298 K in pure water.

**Figure 17:**
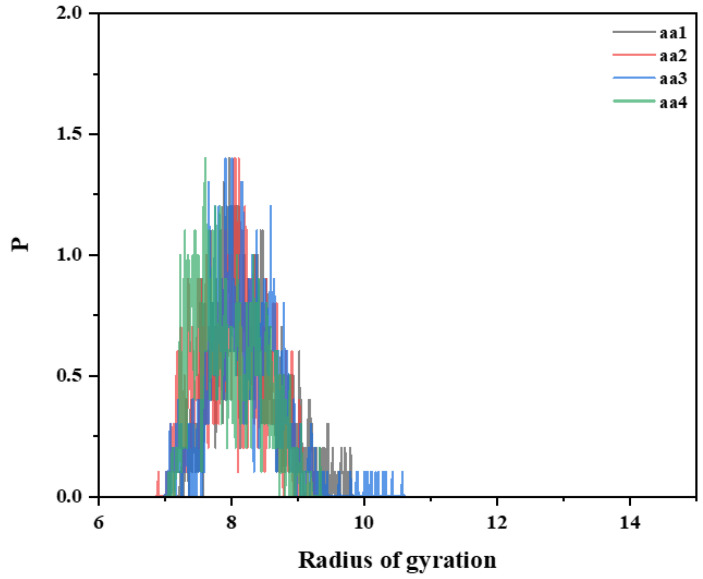
Radius of gyration R_g_ plots of polymer chain segments containing different AA monomers at 473 K in pure water.

The simulation results are shown in Figure 16 and Figure 17. At room temperature, under the same polymerization unit, when the concentration of AA monomers increase, it is beneficial to enhance the intermolecular association and increase the viscosity of the system. When the molar ratio of AA monomers is 30%, the radius of gyration of the chain segments reaches the maximum, and the temperature increase will significantly weaken the promotion of chain stretching by AA monomers. Then, the radius of gyration of the chain segments of the hydrophobically linked polymers becomes smaller, and the polymers with high contents of AA monomers have poorer temperature resistance performance. This can be attributed to the increased thermal movement of the hydrophobic polymer chain segments at high temperatures, resulting in less stretching and intertwining of the molecular chains. This results in freer movement of the polymer chains, which reduces the viscosity of the solution.

## 4. Conclusions

To meet the technical requirements for high-temperature-resistant polymer thickeners in fracturing fluids, this research presents the preparation of a ternary amphiphilic statistical copolymer (MAC) synthesized from the monomers acrylic acid (AA) andacrylamide (AM), along with the hydrophobic monomers. A fracturing fluid system tailored for use in freshwater environments was developed. Using a combination of experiments and molecular dynamics simulations, the conformations and key parameters of the hydrophobic monomers at different temperatures were discussed in depth. The mechanism behind their high-temperature resistance was elaborated, providing a theoretical basis for subsequent polymerizations involving these monomers. The main conclusions are as follows:

1. The results of FTIR and ^1^H NMR confirm the successful synthesis of the ternary hydrophobically linked polymers.
2. The formation of the polymer MAC network structure primarily occurs through the process of contact twist conjugation, with significant intermolecular conjugation observed between 0.2% and 0.4%, consistent with the viscosity–concentration curve results.
3. When 1% of the synthetic polymer was added to form the base fluid, the apparent viscosity of the fluid increased to 133.11 mPa·s, representing a 97% increase in viscosity. The base fluid was crosslinked with 1.5% crosslinker to form a gel. At 90 °C, the controlled loss coefficient (C_3_) and loss velocity (v_c_) of the filter cake were 0.84 × 10^−3^ m/min^1/2^ and 1.40 × 10^−4^ m/min, respectively, both of which met the technical requirements of water-based fracturing fluid.
4. Simulations revealed that the radius of gyration of the hydrophobically linked polymer chain segments decreases as temperature increases. This can be attributed to the increased thermal motion of the hydrophobic polymer chain segments at higher temperatures, which leads to reduced stretching and intertwining of the molecular chains. This results in greater mobility in the polymer chains, which reduces the viscosity of the solution. As a result, the viscosity of hydrophobically bound polymer solutions typically decreases at elevated temperatures.

In conclusion, the fracturing fluid thickener system demonstrates excellent overall performance and significant potential for practical applications. To further promote the field application of hydrophobic association polymer MAC in fracturing fluids, future work will explore the crosslinking mechanism between the polymer and various crosslinkers, as well as its compatibility with other additives.

## Figures and Tables

**Table 1:** Molecular Parameters for the MAC.

Samples	Mw
MAC	1,190,046

**Table 2:** Size of polymer MAC with different concentrations.

Concentration (%)	Particle Size (nm)
0.2	2871
0.4	5811
0.6	7475

**Table 3:** Basic properties of ternary amphiphilic statistical copolymer thickener MAC.

MAC	Characteristic
Solubility	Readily soluble
Solvent	Fresh water (water with low salt content)
Molecular mass	10^5^~10^6^
Temperature resistance (°C)	≥200
Apparent viscosity of 1% polymer solution	133.11 mPa·s

**Table 4:** Apparent viscosity of base solution with 1% ternary amphiphilic statistical copolymer thickener MAC.

Experimental Group	Viscosity Value at the End of Specimen Preparation η_1_/mPa·s	Viscosity Value of the Specimen When Resting for 4 h η_2_/mPa·s
1	128.74	132.61
2	127.84	131.72
3	131.12	134.99
average value	129.23	133.11

**Table 5:** Cumulative filtration losses of 1% polymer MAC crosslinked frozen gel fracturing fluid over time.

Time/s	Cumulative Leachate Loss from Filtrate/mL
1	3.20
2	4.10
4	6.30
9	10.10
16	13.90
25	18.00
36	22.00

**Table 6:** Residue content of fracturing fluid.

Experimental Temperature (°C)	PADM Residue Content (mg/L)
90	28.10

## Data Availability

The original contributions presented in this study are included in the article. Further inquiries can be directed to the corresponding author.
